# Phosducin-like protein *Po*Plp1 impacts cellulase and amylase expression and development in *Penicillium oxalicum* via the G protein–cAMP signaling pathway

**DOI:** 10.3389/fmicb.2023.1165701

**Published:** 2023-06-09

**Authors:** Zhilei Jia, Mengdi Yan, Xiaobei Li, Qiuyan Sun, Gen Xu, Shuai Li, Wenchao Chen, Zhimin Shi, Zhonghai Li, Mei Chen, Xiaoming Bao

**Affiliations:** State Key Laboratory of Biobased Material and Green Papermaking, School of Bioengineering, Shandong Provincial Key Laboratory of Microbial Engineering, Qilu University of Technology, Shandong Academy of Sciences, Jinan, China

**Keywords:** *Penicillium oxalicum*, cellulase, development, G protein-cAMP signaling pathway, phosducin-like protein

## Abstract

In this study, a phosducin-like protein, *Po*Plp1, was identified and functionally studied in the cellulase-producing strain *Penicillium oxalicum* 114-2. *Po*Plp1 was proven to participate in several biological processes, including mycelium development, conidiation, and expression of cellulases and amylases. With deletion of *Po*plp1, morphology and development varied significantly in Δ*Poplp1*. Colony growth, glucose utilization, and the hydrolysis capability of starch and cellulose were limited, whereas conidiation was enhanced. Based on detection of the levels of expression of transcription factors involved in asexual development, we conjectured that *Po*Plp1 is involved in conidiation via the major factor BrlA. We explored the effect of *Po*Plp1 on cellulase and amylase expression and observed that cellulase and amylase activity and major gene transcription levels were all dramatically reduced in Δ*Poplp1*. Deletion of *Po*Plp1 caused a decrease in intracellular cAMP levels, and the cellulase gene expression level of Δ*Poplp1* was restored to a certain extent through external addition of cAMP. These findings demonstrate that *Po*Plp1 may affect cellulase and amylase expression by regulating cAMP concentration. To comprehensively explore the mechanism of *Po*Plp1 in regulating multiple biological processes, we performed a comparative transcriptomic analysis between strains *P. oxalicum* 114-2 and Δ*Poplp1*. The major cellulase and amylase genes were all downregulated, congrent with the results of real-time quantitative polymerase chain reaction analysis. The genes involved in the G protein–cAMP signaling pathway, including several G-protein-coupled receptors, one regulator of G protein signaling, and two cAMP phosphodiesterases, were disrupted by deletion of *Po*Plp1. These results confirm the positive function of *Po*Plp1 in the G protein–cAMP signaling pathway. This functional analysis of *Po*Plp1 will be very beneficial for further study of the regulatory mechanisms of cellulase expression and other biological processes in *P. oxalicum* 114-2 via the G protein–cAMP signaling pathway.

## Introduction

*Penicillium oxalicum* is a filamentous fungus expressing relatively complete and effective lignocellulose-degrading enzymes, which are widely used in various industries, in agriculture, and so on. *P. oxalicum* is also used as a model strain for study of the regulatory mechanisms of cellulase synthesis and asexual development; this research benefits from its mature genetic engineering system. The efficient synthesis of cellulase and several developmental processes (e.g., mycelial growth and conidiation) are co-regulated synergistically by multiple transcription factors in *P. oxalicum* (Li et al., 2015, 2020; Guo et al., 2021; Xu et al., 2022). The regulation of these processes in filamentous fungi is usually triggered by environmental signals (i.e., soluble carbon sources and light), which act on target genes through an intracellular signaling pathway, i.e., the G protein–cAMP signaling pathway (Hu et al., 2013; Cabrera et al., 2022; Schmoll and Hinterdobler, 2022).

The G protein–cAMP signaling pathway is involved in multiple biological processes, such as development, morphogenesis, induced enzyme synthesis, and secondary metabolism (Lafon et al., 2006; Hinterdobler et al., 2020; Cabrera et al., 2022). Heterotrimeric G proteins (consisting of α, β, and γ subunits), G-protein-coupled receptors (GPCRs), regulators of G protein signaling (RGS), the secondary messenger cAMP, and cAMP-dependent protein kinase (PKA) all play pivotal roles in the G protein–cAMP signaling pathway. Phosducin and phosducin-like proteins (PhLPs) are positive regulators of Gβγ heterodimer, and they act as co-chaperones with the cytosolic chaperonin complex for Gβγ folding and assembly in mammals, plants, and fungi (Lukov et al., 2005; Willardson and Howlett, 2007; Castellano and Sablowski, 2008; Gao et al., 2013). Three PhLPs (PhnA, PhnB, and PhnC) are present in *Aspergillus nidulans* (Yu, 2006). PhnA is necessary for Gβγ function. Deletion of *phnA* results in reduced biomass, asexual sporulation in liquid-submerged culture, and defective fruiting body formation; these effects are almost identical to those of the deletion of Gβγ subunits (Seo and Yu, 2006). In *Trichoderma reesei*, PhLP1 (GNB1 and GNG1) also acts as an important node in heterotrimeric G protein signaling, which influences light responsiveness, sexual development, and glycoside hydrolase gene transcription (Tisch et al., 2011). In the plant pathogens *Fusarium graminearum* and *Cryphonectria parasitica*, the putative phosducin-like homolog BDM1 acts on sporulation, germ tube development, and mycelial morphology through the G protein signaling pathway. The virulence of plants has also been found to be significantly reduced after the deletion of BDM1 (Salamon et al., 2010; Horevaj and Bluhm, 2012).

In *P. oxalicum* 114-2, damage to the G protein–cAMP pathway inhibits the growth, development, and expression of amylase and cellulase (Hu et al., 2013); this similarly occurs in other cellulase-producing filamentous fungi, such as *T. reesei* and *A. nidulans* (Amore et al., 2013). In an earlier study, a Gα subunit, PGA3, was found to be essential in the regulatory process for control of the cAMP level in *P. oxalicum* 114-2. However, further regulatory mechanisms and roles of other factors in the pathway have not been confirmed. In order to establish a clear understanding of the effect of the G protein–cAMP pathway on the expression of cellulase and amylase in *P. oxalicum*, we identified a regulatory protein *Po*Plp1 (PDE_07458) in the G protein–cAMP pathway and investigated its roles in regulation of development and cellulase expression.

## Materials and methods

### Strains and culture conditions

The wild-type (WT) strain of *P. oxalicum* 114-2 (CGMCC 5302) was stored in our laboratory. Spores of *P. oxalicum* strains were cultured on potato dextrose agar (PDA) medium at 30°C for 4 days and harvested using sterile water. Counting of the spores indicated that the final concentration was over 10^10^/mL. For mycelium cultivation, 10^8^ spores/mL spores were inoculated in liquid minimal mineral salt (MMS) medium containing 2% glucose and incubated at 30°C and 200 rpm for 24 h. For cellulase production and real-time quantitative polymerase chain reaction (RT-qPCR), exactly 0.5 g pre-cultured mycelia were transferred to 50 mL enzyme production medium (liquid MMS medium containing 1% wheat bran and 1% microcrystalline cellulose). Cellulase production was performed in a 300 mL flask at 30°C and 200 rpm.

### Construction of the deletion and complement strains for *Poplp1*

Deletion of the *Poplp1* gene was performed by the homologous recombination method. The up- and downstream homologous flanking DNA sequences were amplified from the genomic DNA of *P. oxalicum* 114-2 with primers 7458-F1/7458hph-R and 7458hph-F/7458-R1. The hygromycin resistance gene *hph* was amplified from plasmid pSilent-1 using primers hph-F/hph-R. The three fragments were fused by double-joint PCR (Yu et al., 2004), and the full-length deletion cassette was amplified by primers 7458-F2/7458-R2. The deletion cassette was transformed into *P. oxalicum* 114-2 by the polyethylene glycol-mediated method (Li et al., 2010) to obtain the *Poplp1* gene knock-out strain Δ*Poplp1*. *Poplp1* deletion was confirmed using three pairs of primers: primers inside the target gene *Poplp1* 7458-F/R, and primer pairs 7458-F1/hphyz-R and hph-yzF/7458-R1.

For the complement of the *Poplp1* gene, the integrated expression cassette was amplified from the genomic DNA of *P. oxalicum* 114-2 with primers 7458-F1/7458-ptraR. The selected marker gene *ptrA* was amplified from plasmid pME2892 with primers ptrA-F/ptra-7458-R, and downstream homologous flanking DNA sequences were amplified from the genomic DNA of *P. oxalicum* 114-2 with primers 7458-ptraF/7458-R0. The three fragments were fused, and the complement cassette was amplified using primers 7458-F3/7458-R3. The cassette was transformed into the strain Δ*Poplp1* using the abovementioned method, and the complement strain C*Poplp1* was thereby constructed. Complementation of *Poplp1* was confirmed by primers 7458-F/7458-R.

Supplementary Table S1 lists all primers used in the construction of strains Δ*Poplp1* and C*Poplp1*.

### Phenotype analysis

Phenotype analysis was performed on three kinds of sole-carbon-source medium plates (MMS medium containing 2% glucose, 1% cellulose, or 1% starch) and two kinds of compound medium plates (PDA and 10% wheat bran medium) (Li et al., 2015). The plates were inoculated with exactly 1 μL of fresh spores (a total of 10^5^ spores) at a concentration of 10^8^/mL and incubated for 3 days at 30°C.

To measure the biomass of the strains and glucose consumption in the growth medium of the strain, we transferred 0.5 g pre-cultured mycelia to 20 mL of MMS medium containing 2% glucose. All cultures were sampled every 12 h. The mycelia and culture medium were separated by centrifugation and collected separately. Mycelia were dried for 4 h to constant weight at 70°C, and the glucose content of the medium was determined by the 3,5-dinitrosalicylic acid method. Three biological triplicates were performed, and the mean values and corresponding standard deviations were calculated.

The spore production capacity of *P. oxalicum* 114-2 and its mutants was measured on a PDA medium. Exactly 250 μL of fresh spores at a concentration of 10^8^ spores/mL were spread across the plates. The plates were incubated at 30°C, and the number of spores within a fixed area was counted at 24, 30, 36, 48, 60, and 72 h, separately for each strain. Three biological triplicates were performed, and the mean values and corresponding standard deviations were calculated.

To observe the mycelial morphology, we spread 120 μL of fresh spores at a concentration of 10^8^/mL evenly onto PDA plates. Several cover glasses were inserted into the medium slant and the plates were incubated at 30°C. The cover glasses were then removed at 24 and 48 h and observed under an optical microscope at 400× magnification.

### Enzyme activity assay

For the enzyme activity assay, all strains were incubated in the enzyme production medium. Fresh spores were inoculated at 10^8^/mL into liquid MMS medium containing 2% glucose and incubated at 30°C and 200 rpm for 24 h. Next, the mycelia were collected by vacuum pump filtration. Exactly 0.5 g mycelium was transferred to a 50 mL measure of enzyme-producing medium and incubated for 6 days in a 300 mL bottle at 30°C and 200 rpm. Fermentation broths were sampled and measured every 24 h from 72 to 144 h. For the filter paper activity (FPA), endoglucanase, cellobiohydrolase, β-glucosidase, and amylase activity assays, Whatman™ 1 filter paper, sodium carboxymethyl cellulose (CMC-Na, Sigma), *p*-nitrophenyl-β-D-cellobioside (*p*NPC, Sigma), *p*-nitrophenyl-β-D-glucopyranoside (pNPG, Sigma), and starch (Sigma) were used as substrates, respectively (Chen et al., 2013). One unit of enzyme activity was defined as the amount of enzyme required to produce 1 μmol glucose or *p*-nitrophenyl (Sigma) per minute under the assayed conditions (Wood and Bhat, 1988). Three biological triplicates were performed, and the mean values and corresponding standard deviations were calculated.

### RT-qPCR analysis

For RT-qPCR, 0.5 g pre-incubated mycelia of the three strains was induced in 50 mL of cellulase production medium for 4 h. Subsequently, the mycelia were collected for RNA extraction using TRIzol reagent (Invitrogen), in accordance with the operation manual. cDNA and RT-qPCR reaction systems were synthesized using the PrimeScript™ RT reagent kit with gDNA Eraser (Perfect Real Time) and TB Green^®^ Premix Ex Taq™ II (Tli RNaseH Plus) (TAKARA), respectively, following the manufacturers' protocols. The RT-qPCR reaction procedure was performed under the following conditions on a LightCycler^®^480 System (Roche): 95°C for 2 min; 40 cycles at 95°C for 10 s and 61°C for 30 s. The melting curves were measured with a temperature gradient of 0.1°C per second from 65 to 95°C. The expression levels of all genes were calculated using relative quantification, with *actin* as the reference gene. Three biological triplicates were performed, and the mean values and corresponding standard deviations were calculated. Supplementary Table S2 lists the primers used in RT-qPCR.

### Measurement of intracellular cAMP concentration and addition of exogenous cAMP

For each of the three strains, 0.5 g pre-incubated mycelia was cultured in carbon-free MMS medium for 2 h and then transferred to 50 mL of cellulose-inducing medium for 4 and 24 h, respectively. The mycelia were collected by vacuum filtration and ground into powder. The cell contents of 0.1 g of ground mycelia were extracted with 1 mL of HCl (0.1 M). Intracellular protein concentration was determined by the Bradford method (Bradford, 1976); intracellular cAMP concentration was measured by following the instructions of the microbial cAMP enzyme-linked immunosorbent assay kit (Jiangsu Enzyme-Biao Biotechnology Co., Ltd.). The intracellular cAMP concentration for each of the analyzed samples was standardized to the concentration of intracellular protein, with the cAMP concentration of the wild-type strain taken to be 100%. Three biological triplicates were performed, and the mean values and corresponding standard deviations were calculated.

The weighed cAMP powder was added directly to the cellulose induction medium, and 0.5 g hyphae were transferred to the cellulose induction medium with cAMP for 4 h. Samples were collected and their gene expression levels were measured. The expression levels of all genes were calculated using relative quantification, with *actin* as the reference gene. Three biological triplicates were performed, and the mean values and corresponding standard deviations were calculated.

### Transcriptome analysis

For the transcriptome analysis, liquid MMS medium containing 2% glucose was inoculated with spores at 10^8^/mL and incubated at 30°C with agitation at 200 rpm for 24 h. Subsequently, the mycelia were collected by vacuum pump filtration and transferred to a liquid MMS medium without a carbon source. After 2 h of carbon starvation, the mycelia were collected, and 0.5 g mycelia were transferred to 50 mL of liquid MMS medium, including 1% microcrystalline cellulose, and incubated at 30°C with agitation at 200 rpm for 4 h. The RNA of *P. oxalicum* 114-2 and Δ*Poplp1* was extracted using the TRIzol reagent (Invitrogen, USA) in accordance with the manufacturer's protocol. Digital gene expression profiling experiments were performed by Novogene (China) on an Illumina NovaSeq 6000 platform (Illumina, USA). Gene expression levels were indicated by the expected number of fragments per kilobase of transcript sequence per million base pairs sequenced (reads per kb per million reads). The raw RNA-Seq data were deposited at the National Center for Biotechnology Information (NCBI) Sequence Read Archive with the series reference number PRJNA910646. Genes with an adjusted *P*-value ≤0.05 as identified by DESeq2 and fold change ≥ 2 were considered to be differentially expressed. Gene Ontology and Kyoto Encyclopedia of Genes and Genomes enrichment analyses of differentially expressed genes were implemented using the R package clusterProfiler (Yu et al., 2012).

### Statistical analysis

Equal-variance one-tailed *t*-tests were conducted to test comparisons for statistical significance. The mean, corresponding standard deviation, and *P*-value for the corresponding statistical test were calculated in all quantitative analyses. *P*-values≤ 0.05 were considered to represent statistical significance.

## Results

### Sequencing and phylogenetic analysis of *Po*Plp1

**Figure 1:**
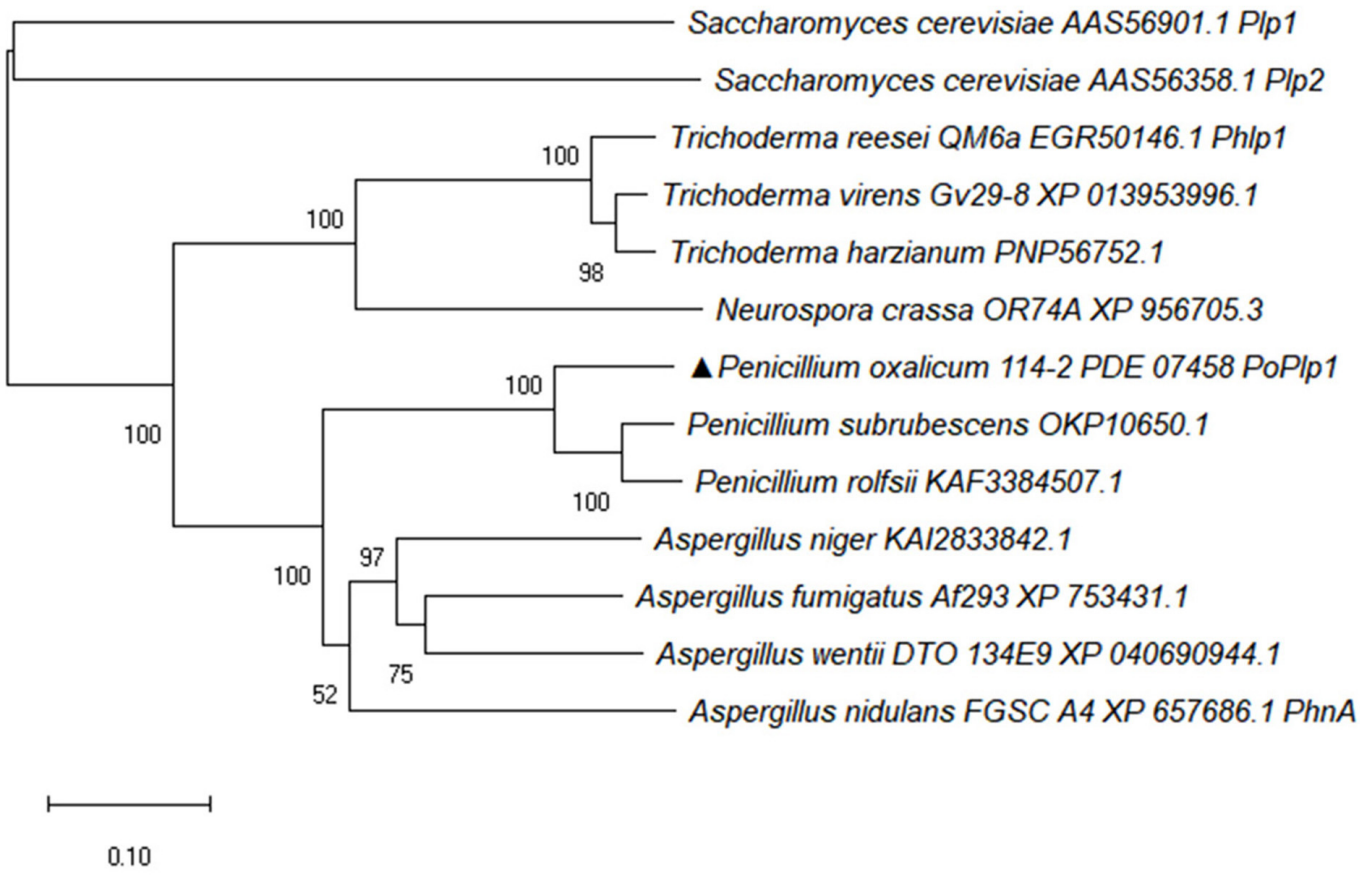
Phylogenetic analysis of PoPlp1. The complete protein sequences of Phlp homolog proteins from *Penicillium* species, *Aspergillus* species, *Trichoderma* species, *N. crassa*, and *S. cerevisiae* were downloaded from the NCBI database. The phylogenetic tree of Phlp homolog proteins was analyzed using the MEGA-X software via the neighbor-joining method.

PhLPs have been identified as downregulators of G protein signaling and they also regulate multiple physiological processes. Here, the PhLP *Po*Plp1 was screened and analyzed in *P. oxalicum* 114-2; it contains a phosducin domain with a length of 159 amino acids (120–159). The complete amino acid sequences of Phlp homolog proteins from *Penicillium* species, *Aspergillus* species, *Trichoderma* species, *Neurospora crassa*, and *Saccharomyces cerevisiae* were downloaded from the NCBI database. The phylogenetic tree of Phlp homolog proteins was analyzed using MEGA-X software via the neighbor-joining method (Figure 1) (Kumar et al., 2018). Sequence alignment of the amino acids of Phlp homologs was completed via the ClustalW multiple alignment function using a biological editing tool. The sequence alignment results showed that the N-terminus and C-terminus of Phlp in *Penicillium* species, *Aspergillus* species, and *Trichoderma* species were highly conserved (Supplementary Figure S1). The amino acid sequence of *Po*Plp1 exhibited the closest relationship with other *Penicillium* species (OKP10650.1, KAF3384507.1), with sequence similarities above 80%. Meanwhile, it shared 56% identity with the homologs of *A. nidulans* FGSC (PhnA, XP_657686.1), which is essential in Gβγ-mediated signaling for the vegetative growth and development of *A. nidulans* (Seo and Yu, 2006). In the case of Phlp1 from *T. reesei* QM6a (EGR50146.1), which influences glycoside hydrolase gene transcription and sexual development, *Po*Plp1 also exhibited 39% identity (Tisch et al., 2011).

In view of the results of sequence alignment, we determined whether *Po*Plp1 played differential roles in regulating growth and development in *P. oxalicum* 114-2.

### *Po*Plp1 functions in development and conidiation in *P. oxalicum* 114-2

**Figure 2:**
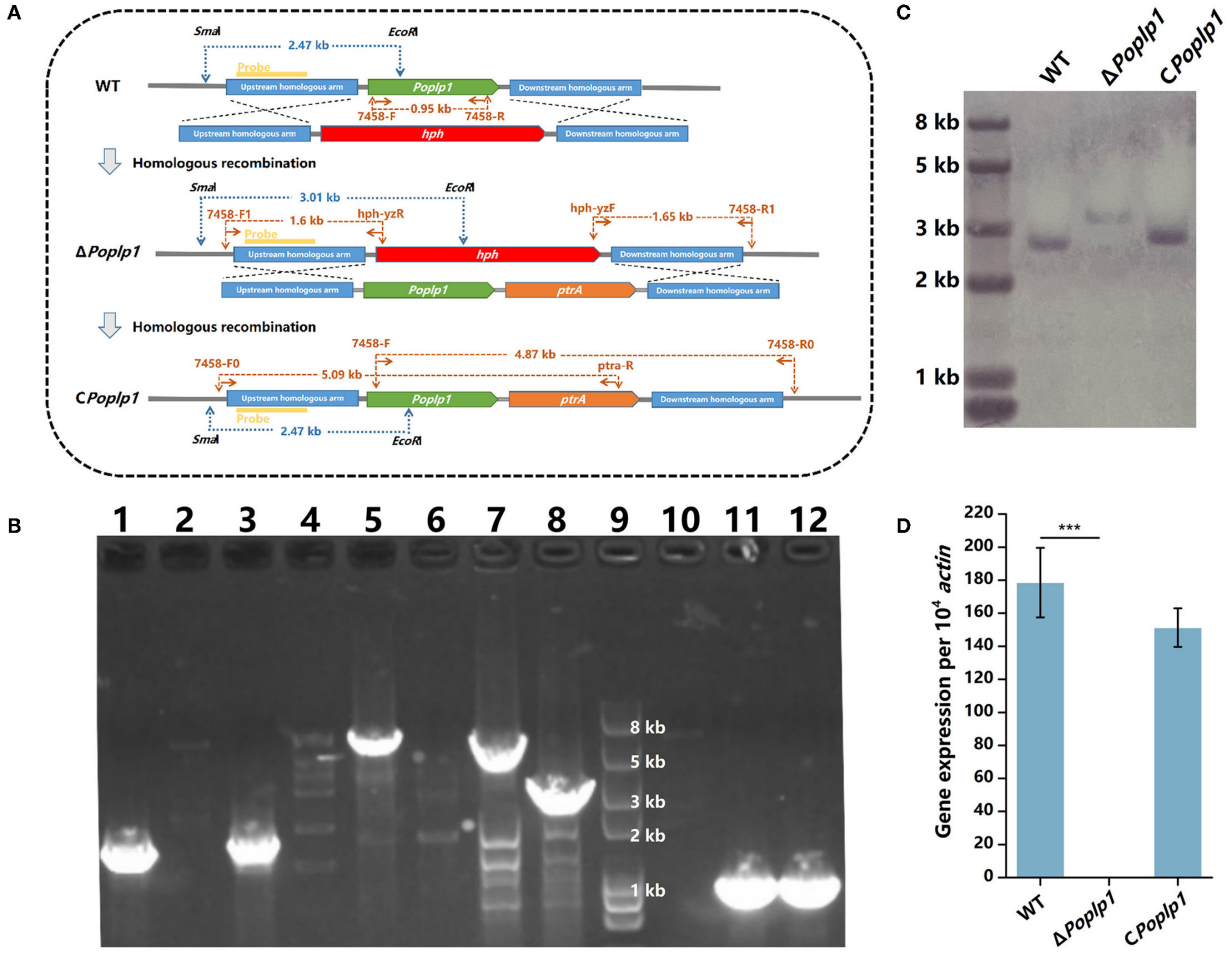
Deletion and verification of *Po*Plp1. **(A)** Strategy for the construction and verification of Δ*Poplp1*. The positions of the primers, probes for Southern blot analysis, and DNA fragment sizes are marked at the corresponding positions. **(B)** PCR analysis of deletion and complementation of *Poplp1*. PCR confirmation of *Poplp1* deletion was performed using three pairs of primers (lanes 1–4); *Poplp1* complementation was confirmed using three pairs of primers (lanes 5–8). (1) Primer pairs 7458-F1/hph-yzR (lanes 1–2) and hph-yzF/7458-R1 (lanes 3–4) (Supplementary Table S1). The theoretical length of the DNA fragment of the deletion strain in each case was 1.60 and 1.65 kb. (2) Primers inside the target gene *Poplp1* 7458-F/R (lanes 10–12). No DNA fragments could be detected in Δ*Poplp1*. PCR complementation of *Poplp1* was verified using primers inside the target gene *Poplp1* 7458-F/R (lane 11). A 0.95 kb-length DNA fragment should be detected. (3) Primer pairs 7458-F0/ptra-R (lanes 5–6) and 7458-F/7458-R0 (lanes 7–8). The theoretical length of the DNA fragment of the complement strain in each case was 5.09 and 4.87 kb. Lanes 2, 4, 6, and 8 are the results for *P. oxalicum* 114-2. Lanes 1, 3, and 10 are the results for *Poplp1*-deletion strain Δ*Poplp1*. Lanes 5, 7, and 10 are the results for the *Poplp1*-complement strain C*Poplp1*. Lane 9 is the Trans2K Plus II DNA marker (TransGen Biotech, China). **(C)** Southern blot analysis of Δ*Poplp1*. The genomic DNA of *P. oxalicum* 114-2, Δ*Poplp1*, and C*Poplp1* was digested by *Sma*I and *EcoR*I. A 0.8 kb-length fragment upstream of the target gene was amplified as the hybridization probe. *P. oxalicum* 114-2 and C*Poplp1* genomic DNA were digested by *Sma*I and *EcoR*I and a 2.47 kb-length fragment containing the complete probe sequence was generated, while a 3.01 kb-length fragment containing the probe sequence was generated from the Δ*Poplp1* genomic DNA. **(D)** qRT-PCR analysis of the gene expression levels of *Poplp1* in the mutant strains. *Poplp1* expression in *P. oxalicum* 114-2, Δ*Poplp1*, and C*Poplp1* was tested using primers RT-7458-F/R inside *Poplp1* (Supplementary Table S2). T-tests were conducted to analyze differences in gene expression levels for significance (^*^P < 0.05).

To confirm the functions of *Po*Plp1 in G protein signaling regulation, we deleted and complemented it in the *P. oxalicum* 114-2 strain through homologous recombination (Figure 2A). Deletion of *Po*Plp1 was verified by PCR, Southern blot, and RT-qPCR (Figures 2B–D).

**Figure 3:**
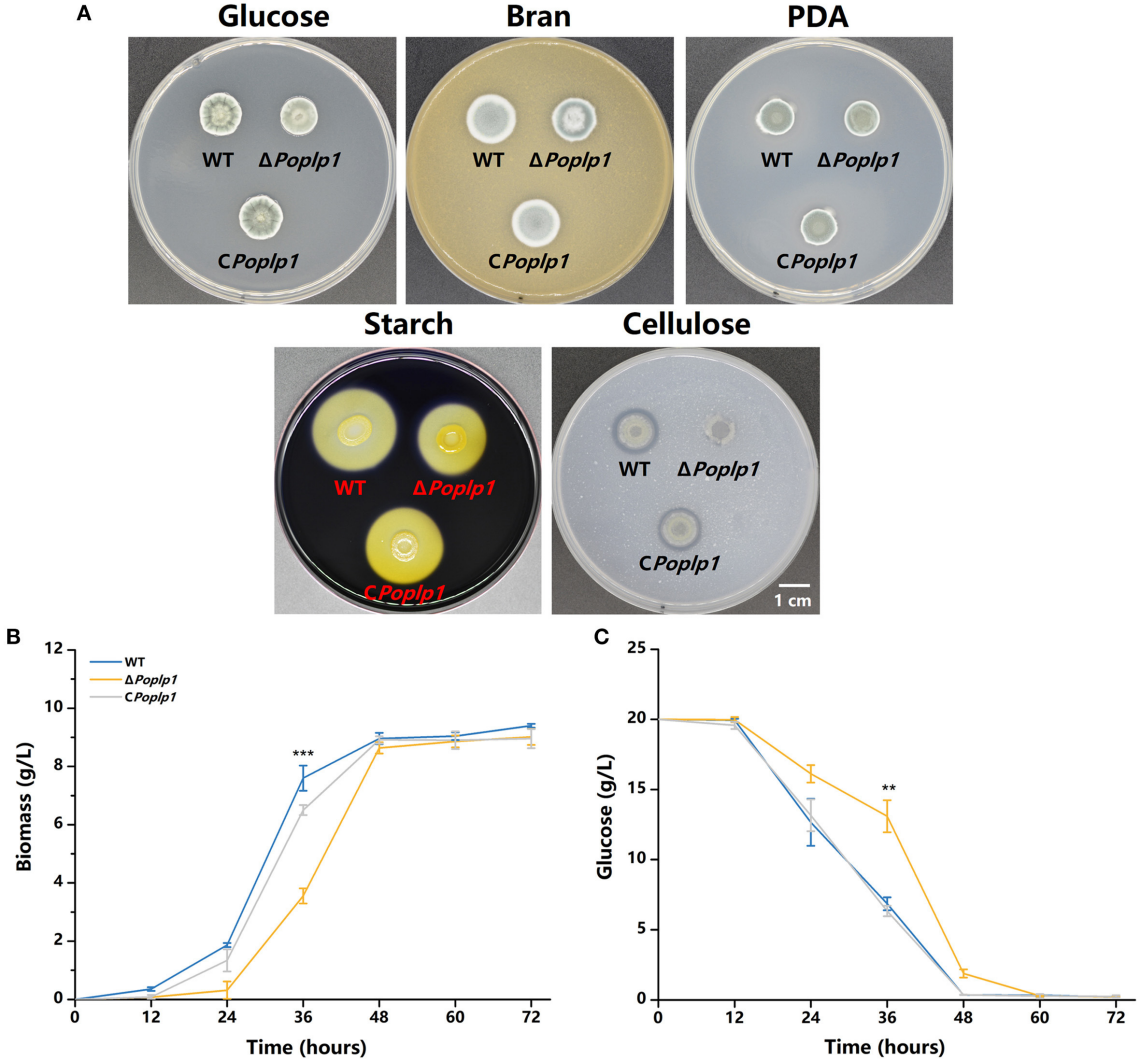
Effect of *Po*Plp1 on the growth and phenotype of *P. oxalicum* 114-2. **(A)** Colony phenotypes of *P. oxalicum* 114-2, Δ*Poplp1*, and C*Poplp1*. Phenotype analysis was performed on MMS medium plates containing 2% glucose, 1% cellulose, or 1% starch, PDA plates, and 10% wheat bran medium plates. Plates were inoculated with 1 μL of suspension liquid at 10^8^ spores/mL and incubated for 3 days at 30°C. **(B)** Effects of the *Poplp1* gene on biomass in glucose culture. The dry weight of mycelia of all strains in the glucose medium was measured every 12 h. **(C)** The rate of glucose consumption by *P. oxalicum* 114-2, Δ*Poplp1*, and C*Poplp1* strains. Residual glucose in the culture medium was detected every 12 h. Three biological triplicates were performed, and the mean values and corresponding standard deviations were calculated; *t*-tests were conducted to analyze the differences in growth and glucose consumption level between *P. oxalicum* 114-2 and Δ*Poplp1* for significance (^*^P < 0.05, ^**^P < 0.01, ^***^P < 0.005).

The influence of *Po*Plp1 on the development of *P. oxalicum* 114-2, including colony phenotype, growth rate, conidiation, and mycelial morphological characteristics, was examined. The colony growth of mutant Δ*Poplp1* on the solid medium containing glucose as the sole carbon source was significantly restricted (Figure 3A), and the colony diameter of Δ*Poplp1* was considerably smaller than that of *P. oxalicum* 114-2. Consistent with this finding, the growth rate of mycelia was also affected by use of a liquid medium with glucose as the only carbon source at 24–48 h (Figure 3B). This outcome may be attributable to deletion of the *Poplp1* gene, which affected the rate of glucose uptake, with a significantly slower rate of glucose consumption observed in the culture medium of Δ*Poplp1* than in that of the original strain at 12–48 h (Figure 3C). In the case of the solid medium with starch or cellulose as the sole carbon source, the diameter of the transparent zone was significantly smaller than in the case of *P. oxalicum* 114-2, which indicates that the hydrolysis abilities of Δ*Poplp1* for starch and cellulose were affected (Figure 3A). Possibly, the lack of *Poplp1* affected the synthesis of starch and cellulose hydrolytic enzymes.

**Figure 4:**
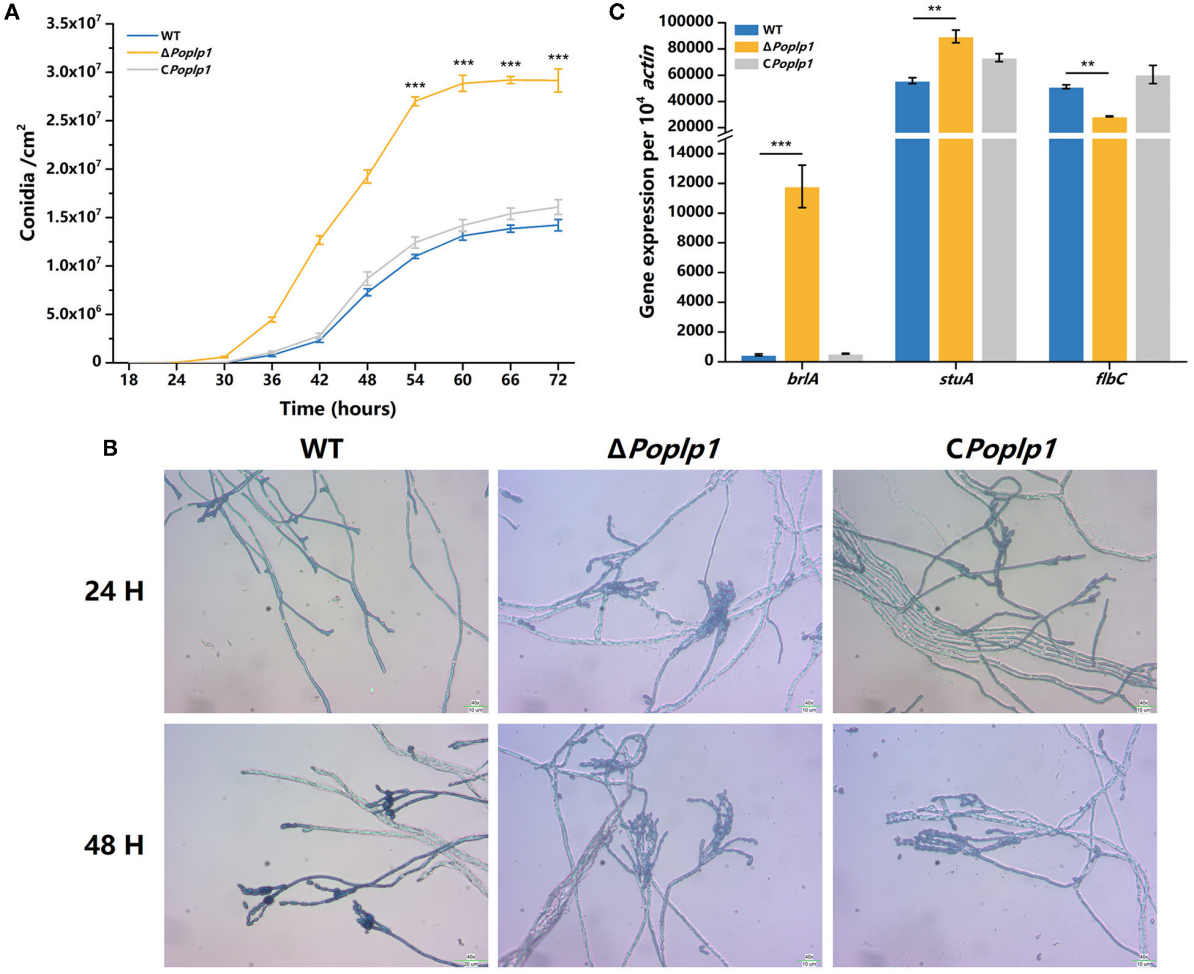
Effect of *Po*Plp1 on conidiation in *P. oxalicum* 114-2. **(A)** Determination of sporulation rate in *P. oxalicum* 114-2, Δ*Poplp1*, and C *Poplp1*. The number of spores within a fixed area of the PDA medium were counted every 6 h. **(B)** Microscopic observations of spore and mycelia morphology on PDA medium. The microscopic morphology of spores and mycelia was observed by optical microscope at 400× magnification after culturing for 24 and 48 h, respectively. **(C)** Analysis of gene expression levels of conidiation-related transcription regulators. The gene expression levels of BrlA, StuA, and FlbC in strains *P. oxalicum* 114-2, Δ*Poplp1*, and C*Poplp1* were detected by RT-qPCR. Three biological triplicates were performed, and the mean values and corresponding standard deviations were calculated. The levels of expression of all genes were calculated using relative quantification, with *actin* as the reference gene; *t*-tests were conducted to analyze the differences in sporulation rate and gene expression level between *P. oxalicum* 114-2 and Δ*Poplp1* for significance (^*^P < 0.05, ^**^P < 0.01, ^***^P < 0.005).

To examine the influence of *Po*Plp1 on conidiation, we measured the conidiation rate of all strains on the PDA plate. Conidiation occurred 6 h earlier for Δ*Poplp1* than for *P. oxalicum* 114-2. At 72 h, the number of conidia of Δ*Poplp1* was twice that of the number for the original strain (Figure 4A). Mycelium morphology at 24 and 48 h indicated that strain Δ*Poplp1* produced conidiophores at 24 h, whereas the parental strain did not (Figure 4B). At 48 h, strain Δ*Poplp1* produced more conidium chains, which resulted in an increase in conidia (Figure 4B).

In *P. oxalicum* 114-2, several key transcriptional regulators of asexual development have been identified; these include BrlA, StuA, and FlbC, which can regulate levels of expression of pigmentation-related and spore wall protein-related genes (Qin et al., 2013; Yao et al., 2016; Li et al., 2019). To explore the mechanism by which *Po*Plp1 affects conidiation, we analyzed the transcription levels of three genes in strains 114-2, Δ*Poplp1*, and C*Poplp1* by RT-PCR. After deletion of *Po*plp1, the levels of expression of *brlA* and *stuA* were significantly enhanced in Δ*Poplp1* and were nearly 25.0- and 1.6-fold higher, respectively, than those occurring in *P. oxalicum* 114-2 (Figure 4C). Meanwhile, expression of *flbC* decreased significantly, undergoing a 44.2% decrease compared with that of *P. oxalicum* 114-2. Given that BrlA is a crucial regulator of conidiation (Qin et al., 2013), we conclude that *Po*Plp1 is involved in conidiation through regulation of transcription levels of the key regulator BrlA.

### Deletion of *Po*Plp1 restricted the expression of cellulases

**Figure 5:**
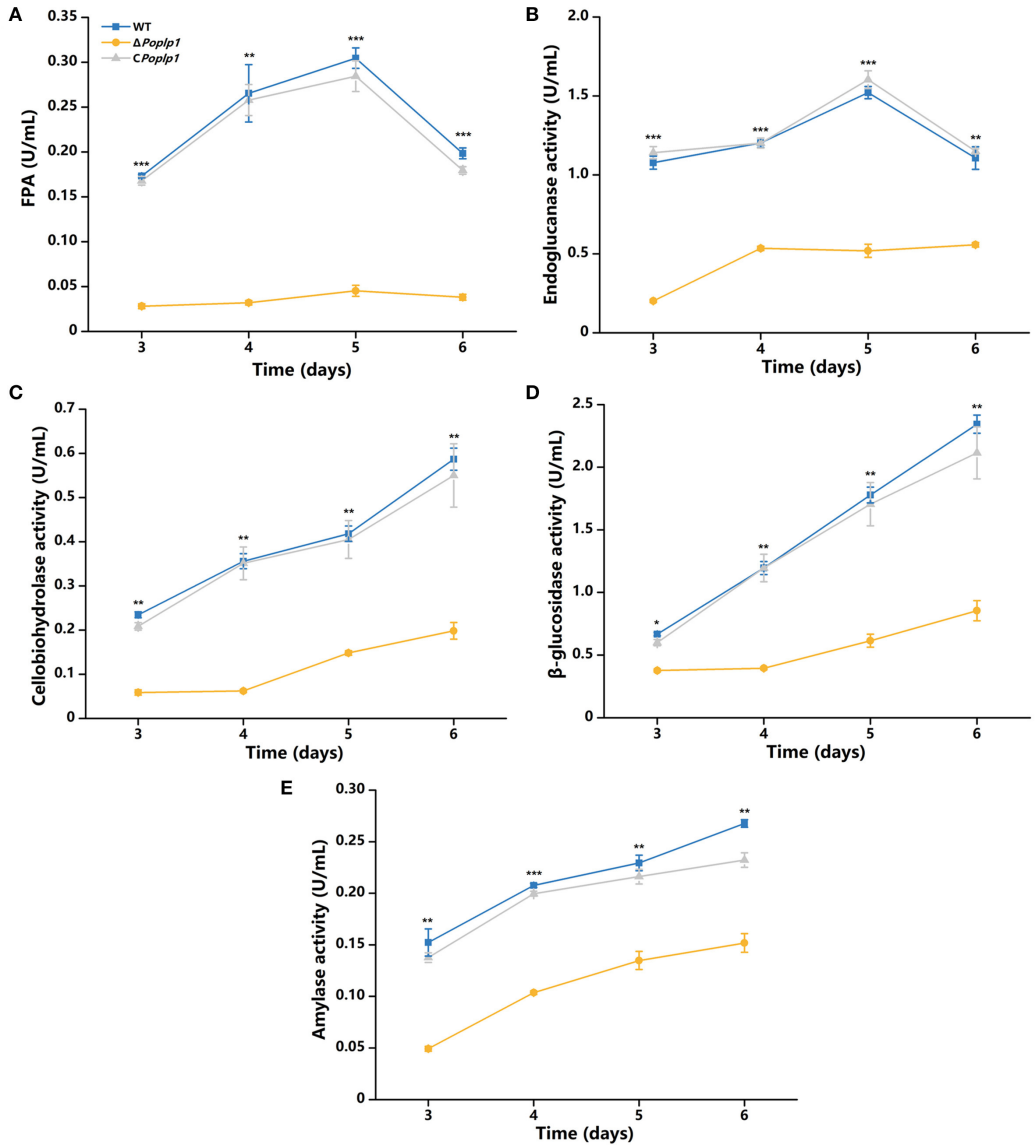
Analysis of the cellulase activity of *P. oxalicum* 114-2, Δ*Poplp1*, and C*Poplp1*. **(A)** FPA; **(B)** endoglucanase activity; **(C)** cellobiohydrolase activity; **(D)** β-glucosidase activity; and **(E)** amylase activity. All strains were sampled and measured every 24 h from day 3 to 6. Three biological triplicates were performed, and the mean values and corresponding standard deviations were calculated; *t*-tests were conducted to analyze differences in the cellulase activity expression levels between *P. oxalicum* 114-2 and Δ*Poplp1* for significance (^*^P < 0.05, ^**^P < 0.01, ^***^P < 0.005).

According to the results of phenotypic analysis of the cellulose medium, *Po*Plp1 affected the synthesis of cellulose hydrolytic enzymes in *P. oxalicum*. Given that *Po*Plp1 acts as a G protein regulator, we focused on the mechanism by which *Po*Plp1 regulates cellulase synthesis in *P. oxalicum*. Its effects on the expression of cellulases were determined by detection of activity toward filter paper (indicating overall cellulase activity), CMC-Na (endoglucanase), *p*NPC (cellobiohydrolase activity), and *p*NPG (β-glucosidase). In Δ*Poplp1*, all detected enzyme activities were reduced. This strain exhibited the highest FPA after fermentation on the fifth day, and its activity was reduced to 15% of that occurring in *P. oxalicum* 114-2 (Figure 5A). Similarly, the endoglucanase activity of Δ*Poplp1* reached its peak on the fifth day and decreased to 34% of that of *P. oxalicum* 114-2 (Figure 5B). The cellobiohydrolase and β-glucosidase activity of Δ*Poplp1* continually increased, with values reaching 34 and 36%, respectively, of those of *P. oxalicum* 114-2 on the sixth day (Figures 5C, D). Activity measurement showed that *Po*Plp1 plays an important role in the production of cellulase, and the absence of *Po*Plp1 dramatically reduced the production of cellulases.

**Figure 6:**
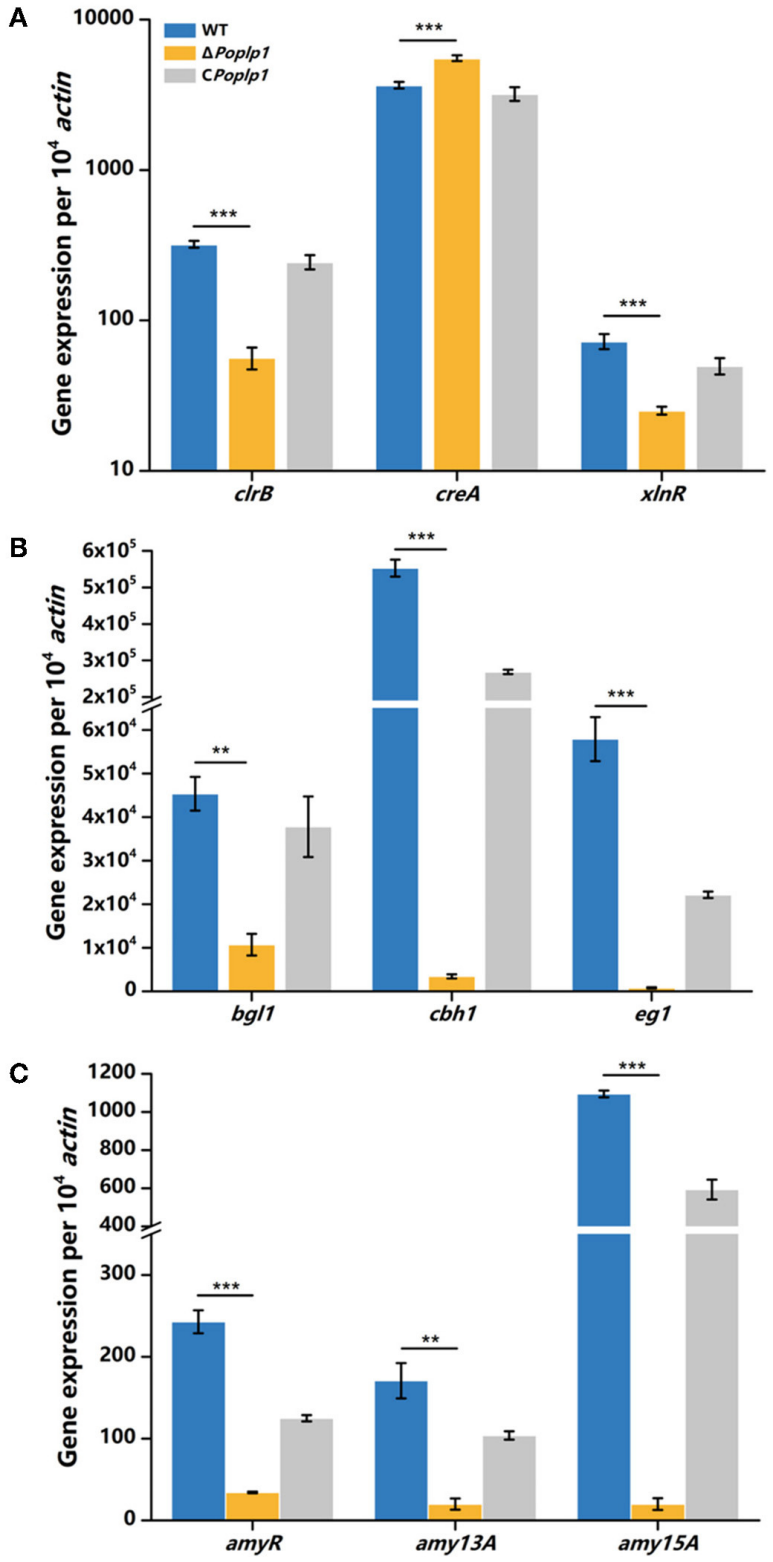
qRT-PCR analysis of the major cellulases and transcriptional regulators of *P. oxalicum* 114-2, Δ*Poplp1*, and C*Poplp1*. Expression levels of **(A)** the four cellulase genes, **(B)** the major transcriptional regulators, and **(C)** amylase genes were measured after induction by cellulose for 4 h. Three biological triplicates were performed, and the mean values and corresponding standard deviations were calculated. The levels of expression of all genes were calculated using relative quantification, with *actin* as the reference gene; *t*-tests were conducted to analyze the differences in gene expression levels between *P. oxalicum* 114-2 and Δ*Poplp1* for significance (^*^P < 0.05, ^**^P < 0.01, ^***^P < 0.005).

To further investigate the mechanism underlying the involvement of *Po*Plp1 in regulating cellulase expression, we analyzed the transcriptional levels of the main cellulase genes and regulators under cellulose induction by RT-qPCR. In Δ*Pohpl1*, expression of the major cellobiohydrolase and endoglucanase genes *cbh1* and *eg1* was substantially reduced, reaching 0.6% and 1.4%, respectively, of the levels of expression observed in *P. oxalicum* 114-2 after inducement by cellulose for 4 h (Figure 6B). The major extracellular β-glucosidase gene *bgl1* in Δ*Poplp1* was also expressed at a lower level than in *P. oxalicum* 114-2; this was reduced by 76.4% in the absence of *Po*Plp1. These results were consistent with the reduction of FPA, cellobiohydrolase, and endoglucanase activity in Δ*Poplp1*, indicating that *Po*Plp1 affects cellulase production at the gene expression level.

Cellulase gene expression was regulated by multiple transcriptional factors in *P. oxalicum* 114-2, including CreA, ClrB, AmyR, and XlnR (Li et al., 2015). CreA and ClrB are the most important transcription repressor and activator of cellulases in *P. oxalicum* 114-2, respectively. AmyR and XlnR are involved in co-regulation of cellulase expression as the major activators of amylase and xylanase, respectively. The RT-qPCR results showed that the gene expression level of CreA increased to 1.5-fold that of *P. oxalicum* 114-2 when induced by cellulose for 4 h, whereas activators ClrB and XlnR decreased to 18 and 34%, respectively, of the levels observed for *P. oxalicum* 114-2 (Figure 6A). Nevertheless, expression of the cellulase gene repressor AmyR decreased noticeably, and was reduced to 14% in *P. oxalicum* 114-2, which was inconsistent with the downregulation of amylase genes *amy13A* and *amy15A* (Figure 6C). We assume that *Po*Plp1 may regulate expression of cellulase and amylase genes in coordination with multiple cellulolytic transcription factors.

### Deletion of *Po*Plp1 reduced intracellular cAMP concentration

**Figure 7:**
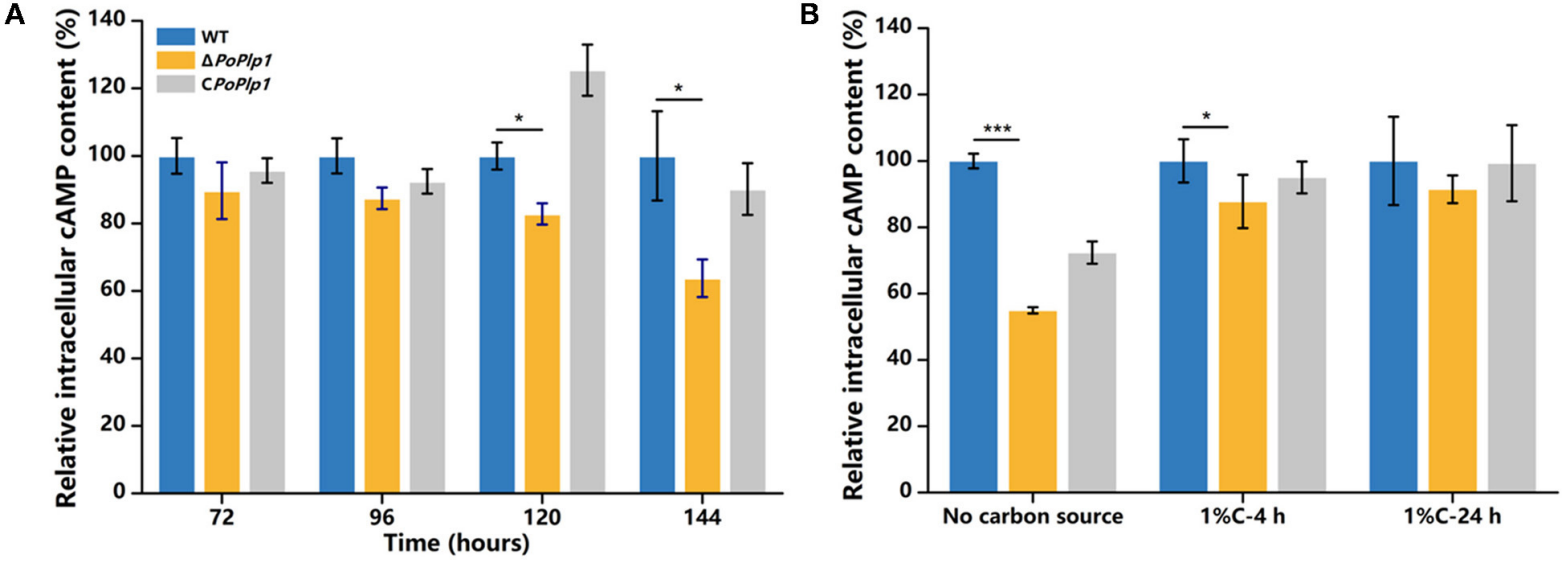
Variation in intracellular cAMP level induced by deletion of *Po*Plp1. **(A)** Intracellular cAMP levels under enzyme production conditions. **(B)** Intracellular cAMP levels under cellulose-inducing conditions. The intracellular cAMP concentrations of all the detected samples were standardized with reference to the same level of intracellular protein. The cAMP concentration of the wild-type strain was considered to be 100%. Three biological triplicates were performed, and the mean values and corresponding standard deviations were calculated; *t*-tests were used to analyze the differences in intracellular cAMP levels between *P. oxalicum* 114-2 and Δ*Poplp1* for significance (^*^P < 0.05, ^**^P < 0.01, ^***^P < 0.005).

Given the role of *Po*Plp1 as a potential G protein regulator and the influence of intracellular cAMP concentration on cellulase and amylase expression in *P. oxalicum* 114-2 (Hu et al., 2013), we hypothesized that *Po*Plp1 regulates cellulase and amylase expression by affecting the levels of expression of transcriptional regulators through the G protein–cAMP signaling pathway. In this study, to investigate the mechanism underlying *Po*Plp1 regulation of cellulase expression, we measured intracellular cAMP levels. When the strains were cultured in the cellulase enzyme production medium, the intracellular cAMP concentration of Δ*Poplp1* was significantly decreased compared with that of *P. oxalicum* 114-2, which was consistent with the decrease in cellulase enzyme activity observed in Δ*Poplp1*. The cAMP concentration in Δ*Poplp1* decreased by 17.2 and 36.2% at 120 and 144 h, respectively (Figure 7A). At the same time, cellulase enzyme activity exhibited the highest levels during this period. Under the cellulose-inducing condition, the intracellular cAMP concentration of Δ*Poplp1* was decreased by 13.3 and by 8.6% after 4 and 24 h, respectively (Figure 7B). This finding was also consistent with the downregulated expression of cellulase genes observed in Δ*Poplp1*. These results showed that *Po*Plp1 regulates the G protein–cAMP signaling pathway by affecting intracellular cAMP levels, which leads to a signal transduction cascade of cellulase and amylase expression.

**Figure 8:**
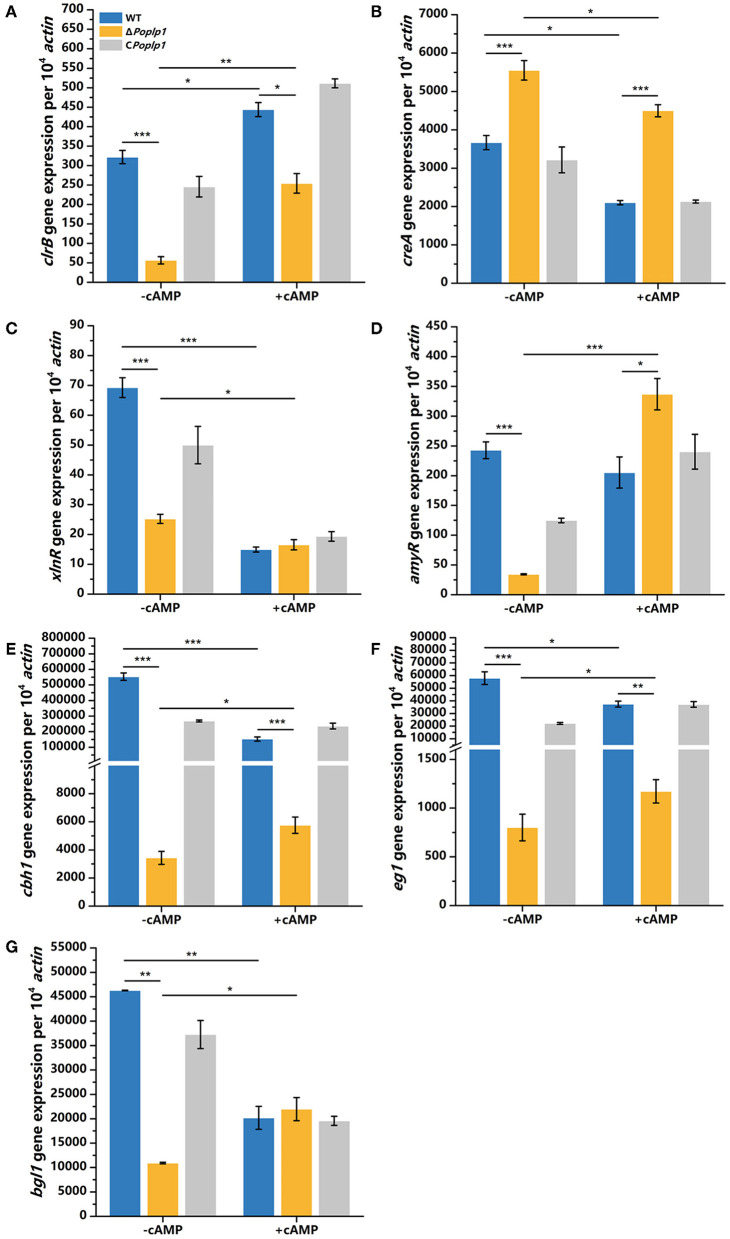
qRT-PCR analysis of the major cellulases and transcriptional regulators of *P. oxalicum* 114-2, Δ*Poplp1*, and C*Poplp1* with the addition of cAMP. Levels of expression of **(A)** *clrB*, **(B)** *creA*, **(C)** *xlnR*, **(D)** *amyR*, **(E)** *cbh1*, **(F)** *eg1*, and **(G)** *bgl1* were observed after induction with 4 mM cAMP cellulose for 4 h. Three biological triplicates were performed, and the mean values and corresponding standard deviations were calculated. The expression levels of all genes were calculated using relative quantification, with *actin* as the reference gene; *t*-tests were conducted to analyze differences in gene expression levels for significance (^*^P < 0.05, ^**^P < 0.01, ^***^P < 0.005).

To further investigate the effect of cAMP on the Δ*Poplp1* cellulase gene and its transcription regulatory factors, we cultured all strains in cellulose medium supplemented with 4 mM cAMP for 4 h and measured the levels of expression of cellulase genes and related transcription regulatory factors. Under this condition, with addition of cAMP, gene expression of the main cellulase transcription activator *clrB* in Δ*Poplp1* was decreased by 42.7% compared with that observed in *P. oxalicum* 114-2; in contrast, without addition of cAMP, gene expression of *clrB* was decreased by 82.4% (Figure 8A). This finding indicates that exogenous addition of cAMP effectively restores expression of the *clrB* gene. The level of expression of the main cellulase transcription inhibitor CreA showed an overall downward trend after the addition of cAMP. However, compared with WT strains, the exogenous addition of cAMP did not effectively restore the gene expression level of CreA (Figure 8B). The gene expression of the other two main transcription factors (XlnR and AmyR) showed two opposing trends (Figures 8C, D). In addition, for Δ*Poplp1*, three major cellulase genes exhibited varying degrees of upregulation after the exogenous addition of cAMP. Moreover, compared with WT strains, their expression levels exhibited varying degrees of recovery (Figures 8E–G). These results indicate that the effect of cAMP on the expression of cellulase genes and their transcription factors is a complex process. However, the addition of cAMP after deletion of *Poplp1* can restore the expression of cellulase genes to a certain extent. This outcome further indicates that downregulation of the cellulase gene in Δ*Poplp1* is caused by a decrease in intracellular cAMP content, which strongly supports the above conclusion.

### Comparative transcriptome analysis

To comprehensively explore the functions of *Po*Plp1, we performed a comparative transcriptomic analysis between strains *P. oxalicum* 114-2 and Δ*Poplp1*. After deletion of *Poplp1*, 158 were genes upregulated and 237 were downregulated. Moreover, the differentially expressed genes were mainly involved in pathways involving the biosynthesis of secondary metabolites (82 genes), starch and sucrose metabolism (23 genes), tyrosine metabolism (17 genes), and phenylalanine metabolism (16 genes) (Supplementary Figure S2).

**Figure 9:**
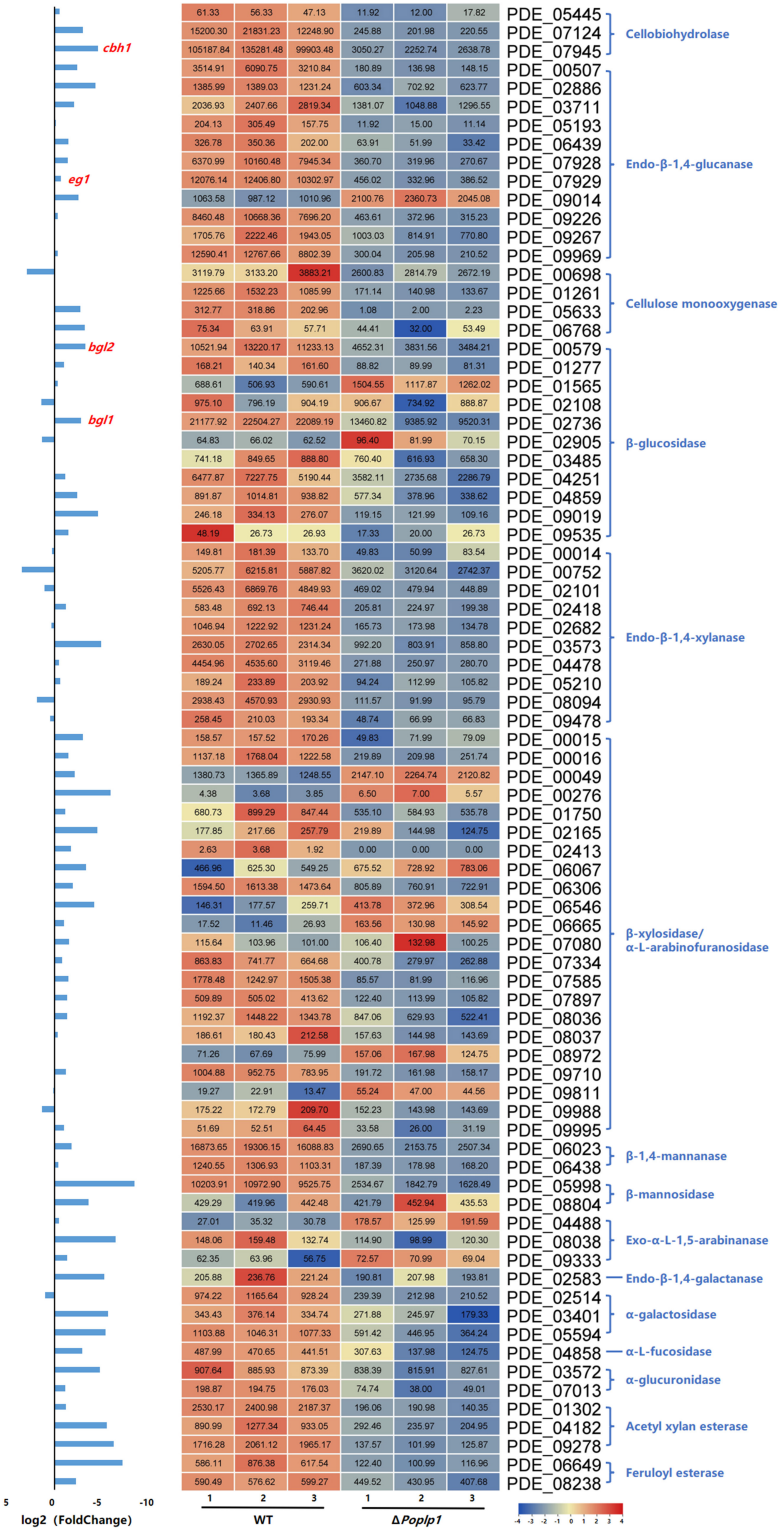
Expression of 80 annotated cellulose hydrolase genes in *P. oxalicum* 114-2 and Δ*Poplp1*. Levels of expression (RPKM) of the 80 cellulose hydrolase genes are indicated by a green–yellow–red color scale. The minimum value is 0, the median is 100, and the maximum value is 8,054.

Considering the regulatory capacity of *Po*Plp1 toward cellulases, the expression levels of 80 annotated cellulolytic genes were analyzed. The results showed that the expression of most cellulase and hemicellulase genes was substantially downregulated in Δ*Poplp1* (Figure 9). Among these genes, the major cellobiohydrolase gene *cbh1*, endo-β-1,4-glucanase gene *eg1*, and extracellular β-glucosidase gene *bgl1* were all dramatically downregulated in Δ*Poplp1*, which was consistent with the results of RT-qPCR.

The genes involved in the G protein–cAMP signaling pathway were analyzed. Although the absence of *Po*Plp1 probably inhibited the activity of G proteins, the gene expression levels of heterotrimeric G proteins (consisting of three Gα, one Gβ, and one Gγ subunits) hardly changed (Table 1). However, the RT-qPCR results showed that deletion of *Poplp1* resulted in significant downregulation of Gβ gene expression levels, which indicates that deletion of *Poplp* may have caused a lack of G beta-folding and a decrease in G protein signaling (Supplementary Figure S3). Meanwhile, the gene expression levels of multiple GPCRs (including one Class III GPCR, two Class IX GPCRs, and several PTH11-like GPCRs) were significantly upregulated in Δ*Poplp1*. This result may be attributable to the interruption of G protein signaling in Δ*Poplp1*, leading to an invalid response by the target genes to external signals. In addition, the expression of GPCRs was upregulated to enhance reception of these signals. Moreover, a Gα regulator, FlbA (PDE_03092), which is also considered to be involved in conidium production (Xie et al., 2022), was dramatically downregulated in Δ*Poplp1*. For the cAMP signaling proteins involved, the transcription levels of adenylate cyclase (PDE_08988) and PKA (catalytic subunit PDE_03213 and regulatory subunit PDE_04688) were barely disrupted by *Po*Plp1. Nevertheless, the expression of a low-affinity cAMP phosphodiesterase (PDE_00536) doubled compared with that observed in the wild-type strain 114-2, while a high-affinity cAMP phosphodiesterase (PDE_02983) was reduced by half in Δ*Poplp1*.

**Table 1:** Transcription levels of genes involving in the G protein–cAMP signaling pathway in *P. oxalicum* 114-2 and Δ*Poplp1*.

**Gene ID**	**Predicted function**	**RPKM** ^*^		**Fold change (Δ*Poplp1*/114-2)**
		**114-2**	Δ***Poplp1***	
**G protein coupled receptors (GPCRs)**				
PDE_02653	Class III GPCR, carbon sensor	17.87	56.78	3.18
PDE_05986	Class IX GPCR	630.07	1939.41	3.08
PDE_04448	Class IX GPCR	0.78	21.62	27.69
PDE_02074	PTH11-like GPCR	8.67	28.77	3.32
PDE_05524	PTH11-like GPCR	8.90	44.37	4.99
PDE_06580	PTH11-like GPCR	7.69	33.57	4.37
PDE_06675	PTH11-like GPCR	0.37	8.47	22.96
PDE_06882	PTH11-like GPCR	0.93	22.23	23.96
PDE_08933	PTH11-like GPCR	8.09	19.96	2.47
PDE_08949	PTH11-like GPCR	25.01	63.40	2.53
**Heterotrimeric G proteins**				
PDE_00146	G protein alpha subunit	266.75	198.01	0.74
PDE_04835	G protein alpha subunit	71.03	61.52	0.87
PDE_00125	G protein alpha subunit	61.83	70.92	1.15
PDE_07459	G protein beta subunit	199.56	161.13	0.81
PDE_02978	G protein gamma subunit	260.61	233.85	0.90
**Regulators of G protein signaling (RGSs)**				
PDE_03092	FlbA (FadA) regulator	34.87	7.96	0.23
PDE_07458	Phosphoducin-like protein *Po*plp1	111.12	0.00	-
**cAMP signaling-involved proteins**				
PDE_08988	Adenylate cyclase	44.17	39.43	0.89
PDE_03213	PKA catalytic subunit	97.27	94.84	0.98
PDE_04688	PKA regulatory subunit	175.46	247.55	1.41
PDE_00536	Low-affinity cAMP phosphodiesterase	20.83	39.82	1.91
PDE_02983	High-affinity cAMP phosphodiesterase	29.50	14.66	0.50

* RPKM, reads per kb per million reads.

The results of this comparative transcriptome analysis provide a comprehensive understanding of the regulatory network involved in *Po*Plp1, which is highly beneficial for study of the regulatory mechanisms of cellulase expression and other biological processes in *P. oxalicum* 114-2.

## Discussion

In numerous species, the G protein–cAMP signaling pathway is involved in multiple biological processes, such as development, morphogenesis, glycoside hydrolase synthesis, and secondary metabolism. A previous study has also confirmed its roles in regulating growth, development, and expression of amylase and cellulase in *P. oxalicum* 114-2 (Hu et al., 2013). However, a comprehensive and deep understanding of the regulatory mechanisms has been lacking. In the present study, a G protein regulator, *Po*Plp1, was identified and functionally studied in *P. oxalicum* 114-2. *Po*Plp1 is conserved with PhLPs in *A. nidulans* and *T. reesei* and is involved in growth, conidiation, sexual development, and glycoside hydrolase gene transcription (Seo and Yu, 2006; Tisch et al., 2011). After deletion of *Po*plp1, phenotypes with respect to these functions were significantly altered in Δ*Poplp1*, including limitation of colony growth and glucose utilization, the hydrolysis capability of starch and cellulose, advancement of conidiation time, and enhancement of conidiation ability and conidium chain (Figures 3, 4). Through detection of the levels of gene expression of several sporulation-related transcription regulators, we observed that expression of the key regulator BrlA, whose absence can completely block conidiation (Qin et al., 2013), was significantly enhanced in Δ*Poplp1*. Therefore, we hypothesize that *Po*Plp1 is involved in conidiation through regulation of the transcription levels of BrlA. Deletion of *Poplp* also led to a decrease in intracellular cAMP content. When cAMP was added to the culture medium, the expression of *brlA* relative to *P. oxalicum* 114-2 did not significantly recover. However, the increase in cAMP content resulted in a joint decrease in the expression of the *brlA* gene in WT and knockout strains (Supplementary Figure S4). This result indicates that the regulation of BrlA by *Po*Plp1 is not caused by changes in intracellular cAMP content, but the level of intracellular cAMP can affect the expression of *brlA*. Another unidentified conidiation regulator, FlbA, was downregulated in Δ*Poplp1* according to the transcriptome analysis (Table 1). FlbA is a negative regulator of G protein; it interacts with Gα subunits and positively modulates conidiophore formation in *Aspergillus* (Yu et al., 1996; Krijgsheld et al., 2013; Xie et al., 2022). However, conidiation was significantly enhanced along with the reduced expression of FlbA in *P. oxalicum* Δ*Poplp1*, which was contrary to the results observed in *Aspergillus*. The function of FlbA in *P. oxalicum* is worth studying to further explain the regulatory mechanism of the G protein–cAMP signaling pathway in development.

Based on the phenotype of reduced hydrolysis capability for starch and cellulose in Δ*Poplp1*, the activities of FPA, cellobiohydrolase, endoglucanase, β-glucosidase, and amylase were all dramatically decreased (Figure 5). Most of the annotated glycoside hydrolase genes, including *cbh1, eg1*, and *bgl1*, were also downregulated in Δ*Poplp1*, according to the transcriptome analysis (Figures 6, 9). After deletion of *Poplp1*, expression of the main cellulase transcription activator *clrB* was significantly downregulated, whereas that of the main cellulase transcription inhibitor *creA* was significantly upregulated (Figure 6). This finding indicates that changes in the levels of expression of transcription factors may be the direct cause of the reduced gene expression of cellulase and amylase. The regulation of the G protein–cAMP signaling pathway to cellulase and amylase genes is carried out via control of cAMP levels (Hu et al., 2013). The results of the measurement of intracellular cAMP showed that deletion of *Po*Plp1 caused a decrease in intracellular cAMP levels of Δ*Poplp1* (Figure 7). When cAMP was added to the culture medium, the level of expression of the main cellulase transcription activating factor ClrB was significantly restored, and those of the main cellulase genes also exhibited varying degrees of recovery (Figure 8). This finding indicates that the decrease in the levels of cellulase gene expression caused by *Poplp1* deficiency can be partially restored by the exogenous addition of cAMP. This result confirmed the positive function of *Po*Plp1 in the G protein–cAMP signaling pathway, and indicated that *Po*Plp1 may affect cellulase and amylase expressions by regulating the cAMP levels.

To comprehensively explore the mechanisms underlying the involvement of *Po*Plp1 in the regulation of multiple biological processes, we performed a comparative transcriptomic analysis between strains *P. oxalicum* 114-2 and Δ*Poplp1*. The genes involved in the G protein–cAMP signaling pathway were analyzed. The results indicated that *Po*Plp1 may function in the G protein–cAMP signaling pathway by influencing the activity of G proteins, rather than their expression levels. The gene expression levels of heterotrimeric G proteins (consisting of three Gα, one Gβ, and one Gγ subunits) hardly changed (Table 1). However, the RT-qPCR results showed that the deletion of *Po*plp1 resulted in significant downregulation of Gβ gene expression (Supplementary Figure S3), which implies that deletion of Poplp may have caused a deficiency in G beta-folding and a decrease in G protein signaling. GPCRs are important components for signal reception and the main upstream components of the G protein signaling pathway (Syrovatkina et al., 2016). In the transcriptome data, several GPCRs (including one Class III GPCR, two Class IX GPCRs, and several PTH11-like GPCRs) exhibited significantly enhanced expression in Δ*Poplp1*, which enhances signal reception in cases of G protein signaling obstruction. The negative RGS FlbA binding to Gα was dramatically downregulated in Δ*poplp1*, which increased the G protein activity by reducing the level of expression of Gα inhibitory factors. Given that the downstream G protein signaling pathway directly acts on adenylate cyclase (Sun et al., 2022), the upstream signal of G protein was blocked, which generally leads to a chain reaction downstream. However, the transcription level of adenylate cyclase did not change significantly, whereas that of intracellular cAMP decreased significantly. Therefore, the deletion of *Po*Plp1 does not affect the transcription level of adenylate cyclase, but does affect the synthesis of intracellular cAMP by adenylate cyclase. Reduction of the cAMP level will affect the regulation of multiple downstream biological pathways through a change in the target protein phosphorylation level (Sun et al., 2022). Nevertheless, the expression of a low-affinity cAMP phosphodiesterase (PDE_00536) was doubled compared with that observed in WT strain 114-2, whereas a high-affinity cAMP phosphodiesterase (PDE_02983) was reduced by half in Δ*Poplp1*. This may be attributable to mechanisms operating to maintain the levels of secondary messengers (i.e., cAMP and cGMP) to ensure the necessary physiological regulation (Azevedo et al., 2014). The comparative transcriptome results provide a comprehensive picture of the regulatory network with which *Po*Plp1 is involved, which will helpful in further investigation of the functions of *Po*Plp1 and the G protein–cAMP signaling pathway.

In this study, a G protein-positive regulator, *Po*Plp1, was identified and functionally studied in *P. oxalicum* 114-2. *Po*Plp1 was found to participate in several biological processes, including mycelium development, conidiation, and the expression of cellulases and amylases. The findings of this study are very beneficial for further study of the regulatory mechanisms underlying cellulase expression and other biological processes in *P. oxalicum* 114-2 via the G protein–cAMP signaling pathway.

## Data availability statement

The datasets presented in this study can be found in online repositories. The names of the repository/repositories and accession number(s) can be found in the article/Supplementary material.

## Author contributions

ZL conceived and designed the experiments. ZJ, MY, XL, QS, GX, SL, and WC performed the experiments. ZJ, MC, and ZS analyzed the data. MC and ZL drafted the manuscript. XB revised the manuscript. All authors read and approved the final manuscript.
