# Supplementary material for: Phosducin-like protein PoPlp1 impacts cellulase and amylase expression and development in Penicillium oxalicum via the G protein–cAMP signaling pathway
Source: Front Microbiol. 2023 Jun 9;14:1165701. doi: 10.3389/fmicb.2023.1165701 (PMC10289023; doi:10.3389/fmicb.2023.1165701)
Supplement: Supplementary file 1 [file Data_Sheet_1.docx]

Supplementary Material

Phosducin-like protein PoPlp1 impacts cellulase and amylase expression and development in *Penicillium oxalicum via* the G protein-cAMP signaling pathway

**Zhilei Jia^1^, Mengdi Yan^1^, Xiaobei Li^1^, Qiuyan Sun^1^, Gen Xu^1^, Shuai Li^1^, Wenchao Chen^1^, Zhimin Shi^1^, Zhonghai Li^1^*, Mei Chen^1^*, Xiaoming Bao^1^**

*** Correspondence:**

Zhonghai Li:lzhlzh@vip.126.com

Mei Chen:chenmei_16@163.com


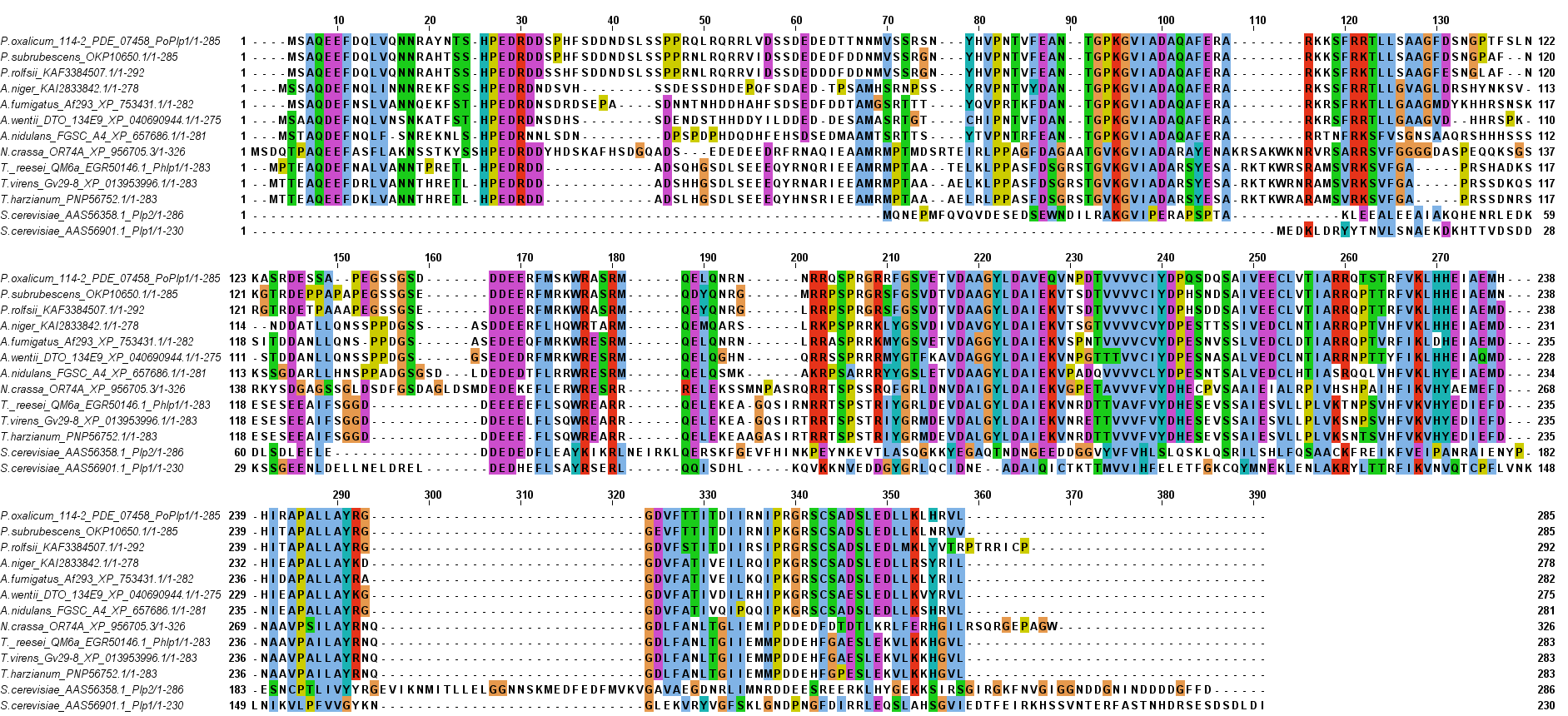


**Supplementary Figure 1. Sequence alignment of the Phlps homolog homeodomains.** The alignment was performed by the ClustalW Multiple Alignment function in the Bioedit tool.


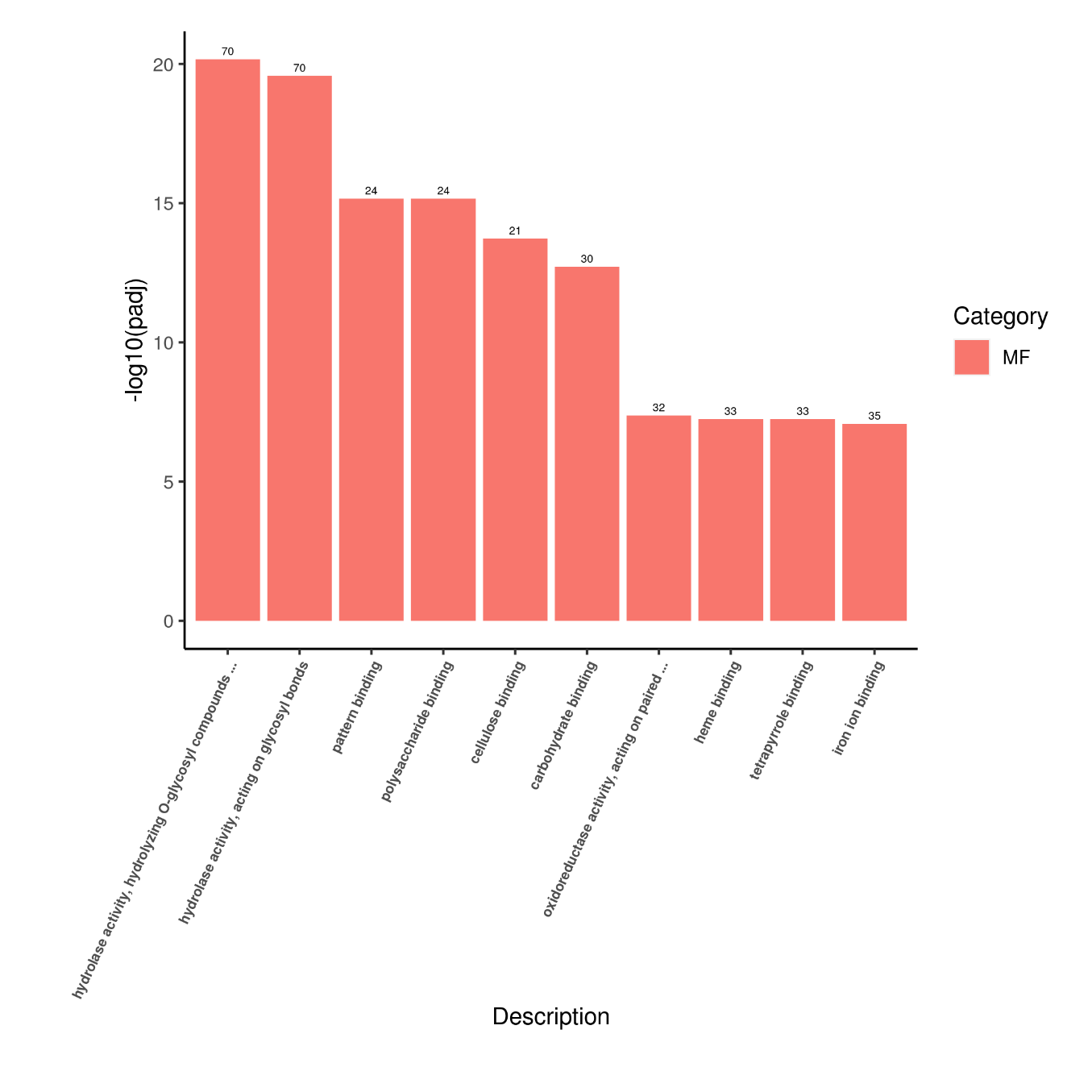


**Supplementary Figure 2.** **GO enrichment analysis of the differential expressed genes of *P. oxalicum* 114-2 and Δ*Poplp1*.**

**
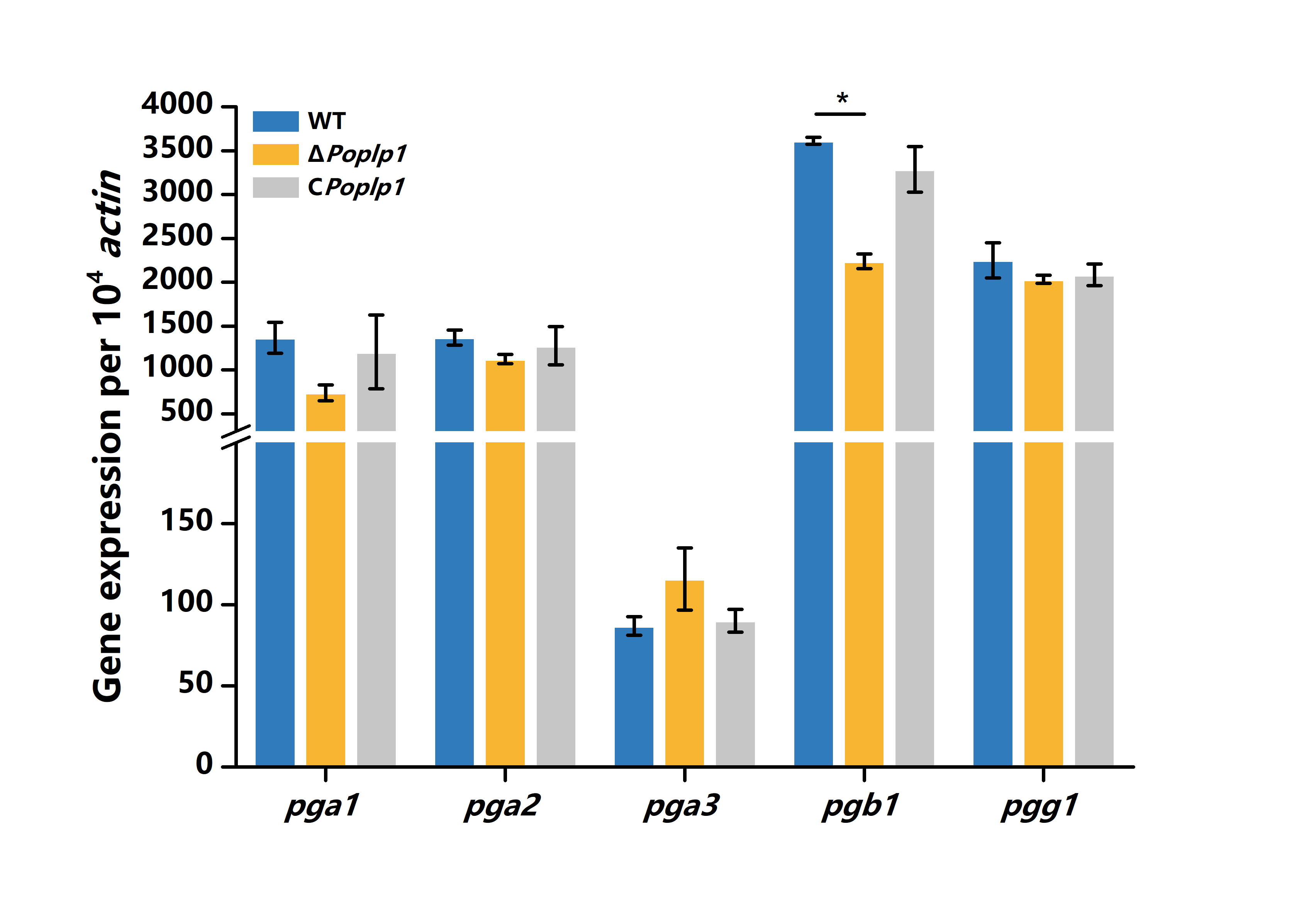
**

**Supplementary Figure 3. qRT-PCR analysis of G alpha, G beta and G gamma of P. oxalicum 114-2, Δ*Poplp1* and C*Poplp1*.** Measure the expression level of G alpha, G beta and G gamma gene after 4 hours of cellulose induction. The *t*-test was used to analyze the significant differences (*P < 0.05, **P < 0.01, ***P < 0.005) of gene expression levels between *P. oxalicum* 114-2 and Δ*Poplp1*.


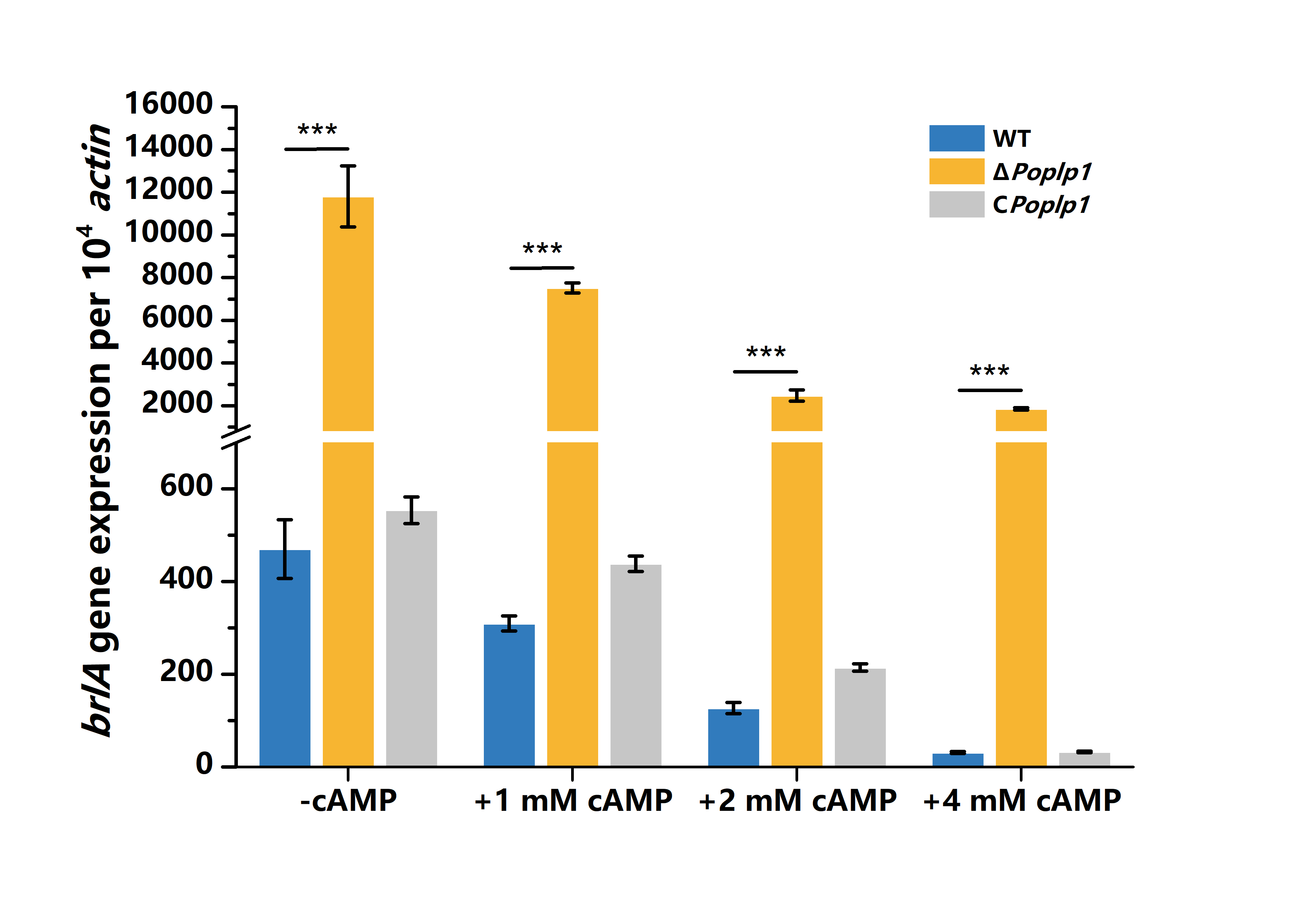


**Supplementary Figure 4. qRT-PCR analysis of *brlA* of P. oxalicum 114-2, Δ*Poplp1* and C*Poplp1* with the addition of cAMP.** After 4 hours of cultivation in cellulose induced medium with different concentrations of cAMP added, the expression level of brlA gene was measured. The *t*-test was used to analyze the significant differences (*P < 0.05, **P < 0.01, ***P < 0.005) of gene expression levels between *P. oxalicum* 114-2 and Δ*Poplp1*.

**Table S1. Primers used for construction and verification of *P. oxalicum* Δ*Poplp1* and C*Poplp*.**

| **Primers** | **Primer Sequences（5'-3'）** |
| --- | --- |
| **Primers used for construction and verification of Δ*Poplp1*** | |
| hph-F | CGACGTTAACTGATATTGAA |
| hph-R | CAACCCAGGGCTGGTGACGG |
| 7458-F1 | TCTATCTGCCGGTCCTTTAT |
| 7458-hphR | GCTCCTTCAATATCAGTTAACGTCGGAAGGGTTTAAAGATGACGG |
| 7458-hphF | AAATTCCGTCACCAGCCCTGGGTTGTTCCCTCTGATCTCGTTCTA |
| 7458-R1 | GTGGTTCCTTCTCGACCATG |
| 7458-F2 | TTCCACTTTCTCACCGTTTC |
| 7458-R2 | CTTTGAGAATGTCCAGTGCC |
| hph-yzF | GCGCCGCCGCTACTGCTTAC |
| hph-yzR | ACTGAGGAATCCGCTCTTGG |
| 7458-F | TGCCTACAATACCTCCCATCC |
| 7458-R | GGAGGCACGTACAGTTTCA |
| **Primers used for construction and verification C*Poplp1*** | |
| ptra-F | GGGCAATTGATTACGGGATC |
| ptra-R | ATGGGGTGACGATGAGCCGC |
| 7458-F1 | TCTATCTGCCGGTCCTTTAT |
| 7458-ptraR | GGATCCCGTAATCAATTGCCCTGTGAGAATGGACCAGCAA |
| ptra-7458R | TACTTAGGAGAGGGTGGCACTATATATGGGGTGACGATGAGCCGC |
| 7458-ptraF | ATATAGTGCCACCCTCTCCTAAGTA |
| 7458-R0 | ATGAGCCATTGCTGACCAC |
| 7458-F3 | TTCCACTTTCTCACCGTTTC |
| 7458-R3 | CGACTGACATTTGGAACCC |

**Table S2. Primers used in RT-qPCR.**

| **Primers** | **Primer Sequences（5'-3'）** |
| --- | --- |
| RT-act-F | GTTCCATTCTCGCCTCCCTCT |
| RT-act-R | AGAAGCACTTGCGGTGAACGA |
| RT-cbh1-F | CCACCACCACTACCAGCAAGG |
| RT-cbh1-R | GTAGCCAACACCACCGCACT |
| RT-eg1-F | ACCGCTGCTCAGACCACGAC |
| RT-eg1-R | TGGGTCCCGAGTAGCCAACG |
| RT-bgl1-F | CACCAACACCGGCTCAGTTA |
| RT-bgl1-R | GGACATCCCAGTTGGACAGAT |
| RT-bgl2-F | GGCTGATGCGTACACGTTTGA |
| RT-bgl2-R | CGACATAAGTCACGCCGAAGC |
| RT-creA-F | ACAGTCCTGGTCAAGGTCAC |
| RT-creA-R | GCCCGCCACGGAATTATTTG |
| RT-clrB-F | TTGCCCGCATTTACGAAGCC |
| RT-clrB-R | GTCTTGGGGTCCATTTTCGC |
| RT-xlnR-F | GTGGTCCGAGCCTGCGAAAC |
| RT-xlnR-R | CAGCGGTAGAGGGCGAGAAC |
| RT-amyR-F | CCATCGGCAACTTTCTCCCA |
| RT-amyR-R | TGAATGCGCTCGTGATGCTC |
| RT-amy13A-F | CTAATGCCGTTGCGTTGTCC |
| RT-amy13A-R | TTTCCCAAGTGACCGAGCC |
| RT-amy15A-F | CACTCCTGACTGCCAACGAC |
| RT-amy15A-R | CCAACAGAAGGAGTGGCGTA |
| RT-brlA-F | GGAACATCTCAAGCGGCACA |
| RT-brlA-R | CAACTTGGAGCCGTAGATGG |
| RT-flbC-F | TACGGGTTCCCTACCAATCA |
| RT-flbC-R | GAATTGGCTCATCATGGACTG |
| RT-stuA-F | GCGGCTCCTACACTTACACC |
| RT-stuA-R | GAACGGCATCGTCGTCATCG |
| RT-7458-F | CTCCACCAATGTCCGCACCA |
| RT-7458-R | ACGGTGACGCAGAGGCAGTA |
| RT125-F | CGGAACAGAAGAAGAGGAGC |
| RT125-R | GCTCTTCCACGGTGTAGCC |
| RT146-F | GCAACACCGTCCAGTCTATG |
| RT146-R | GAAGATAGTCTGGACGTGGTAC |
| RT2978-F | TGTCGCCGACCTCAAGTAT |
| RT2978-R | AGCCTCCGAGACCTTCACT |
| RT4835-F | TCATTCACAGCGGAGGGTT |
| RT4835-R | CGCTTCGGTGCCTTCTAAC |
| RT7459-F | CGTGGCTTGCGGTGGTCTT |
| RT7459-R | GATGAAGCGGCAGCAGGAC |
